# The effects of cyberbullying victimization on cyberbullying perpetration among Chinese college students: callous-unemotional traits and the moderating role of Internet morality

**DOI:** 10.3389/fpsyg.2024.1326237

**Published:** 2024-04-03

**Authors:** Wenhai Zhang, Jingying Sha

**Affiliations:** ^1^Graduate School, People’s Public Security University of China, Beijing, China; ^2^Faculty of Criminology, People’s Public Security University of China, Beijing, China

**Keywords:** cyberbullying victimization, cyberbullying perpetration, callous-unemotional traits, Internet morality, co-moderating

## Abstract

**Introduction:**

The Internet has triggered a series of online deviant behaviors, and cyberbullying is one of them. Cyberbullying victimization as a category of frustration and the aggression triggered by it has been confirmed by many studies. Previous studies have explored the relationship between cyberbullying victimization and cyberbullying perpetration. However, the boundary conditions of the two have yet to be sufficiently explored, and this article will further explore the moderating effect in the transformation mechanism.

**Methods:**

The convenience sampling method was used to select a cumulative total of 668 students from university students of several universities in Beijing for the study, using questionnaires including Cyberbullying Victimization Questionnaire, Cyberbullying Perpetration Questionnaire, the Callous-unemotional Traits Scale, and Internet Morality Questionnaire.

**Results:**

(1) Controlling for gender and grade, cyberbullying victimization has a positive relationship with cyberbullying perpetration. (2) Callous-unemotional traits moderated the relationship between cyberbullying victimization and perpetration. (3) Internet morality can moderate the relationship between cyberbullying victimization and perpetration. (4) Callous-unemotional traits and Internet morality can co-regulate the relationship between cyberbullying victimization and perpetration.

**Conclusion:**

The results indicate that cyberbullying victimization had a significant positive relationship with cyberbullying perpetration, a process moderated by callous-unemotional traits and Internet morality.

## 1 Introduction

With the development of information and communication technology, more and more people have joined the “Internet army” for learning, working, and socializing. The virtual nature, anonymity, and even the arbitrariness of moral norms of the Internet have thus triggered a series of online deviant behaviors, and cyberbullying has a high incidence among Chinese Internet users (Zhong et al., 2021). Adolescents are a susceptible group to cyberbullying (Bauman et al., 2013). However, as one of the leading forces of Internet use (China Internet Network Information Center [CINIC], 2023), college students in the youth group use the Internet much more frequently than primary and secondary school students. Suppose college students feel cyberbullying by outgroups when using social media. In that case, their emotional feelings will be rendered and incited by each other, which will lead to negative emotions such as aggrievement, anxiety, depression, and even self-harm or suicidal behaviors (Iranzo et al., 2019). The study of college students’ cyberbullying has increasingly become a focus of attention for researchers in psychology (Gosling and Mason, 2015). Kim and Lee (2023) point out that a person may be motivated to cyberbully another person due to being cyberbullied. In criminology, the process of reverse malignant transformation from victim to perpetrator, driven by lousy psychology, is called “evil reversal” (Guo, 1997). The phenomenon of “evil reversal” also exists in cyberspace. This article intends to take cyberbullying victimization as the independent variable to explore the influence and mechanism of cyberbullying perpetration.

### 1.1 Cyberbullying victimization and cyberbullying perpetration

Cyberbullying perpetration is the intentional and repeated infliction of harm on another person via electronic media (Patchin and Hinduja, 2006). According to the frustration-aggression hypothesis (Gilbert and Bushman, 2020), frustration in life experiences can trigger various aggressive behaviors. Cyberbullying victimization as a category of frustration and the aggression triggered by it has been confirmed by many studies (Zhu et al., 2019; Wang Q. Q. et al., 2020; Xia and Sha, 2021). A study on the effects of adolescent cyberbullying victimization on deviant behavior showed that cyberbullying victimization positively predicts deviant behavior (Li et al., 2015; Wang Y. L. et al., 2020). Meanwhile, a meta-analysis on cyberbullying perpetration showed a positive correlation between cyberbullying victimization and cyberbullying perpetration (Kowalski et al., 2014). It has also been shown that among adolescents, there is a joint trajectory between cyberbullying victimization and cyberbullying perpetration (Camacho et al., 2023), and cyberbullying victimization can unidirectionally predict cyberbullying perpetration (Wang, 2020). Previous studies have explored the relationship between cyberbullying victimization and cyberbullying perpetration. However, not all cyberbullying victims would become perpetrators, and the influence processes between them need to be further explored. This article will further explore the moderating effect of the transformation mechanism.

### 1.2 The moderating role of callous-unemotional traits

The general model of aggression states that aggression cannot occur without a combination of the individual and the environment (Anderson and Bushman, 2002). This article will explore the moderating roles of personality dispositions and subjective norms in the predictive roles of cyberbullying victimization on cyberbullying perpetration from the individual’s perspective. As an essential input variable in individual factors, personality traits significantly predict aggressive behavior (Anderson and Bushman, 2002). For instance, loneliness positively predicts cyberbullying victimization and cyberbullying perpetration (Zhou et al., 2019). The callous-unemotional trait is a personality trait that treats others with indifference, lacks culpability and empathy, and is characterized by stability, severity, and ease of aggression (Xiao et al., 2014). Most of the studies that have been conducted have used callous-unemotional traits as a predictor, such as being able to predict bullying behavior positively (Zhang et al., 2023) and cyberbullying perpetration (Fang and Wang, 2020). However, as a personality trait-like variable, the callous-unemotional traits are usually moderate. Some empirical studies have shown that callous-unemotional traits can effectively modulate the effects of adverse life experiences on college students’ consciences (Wang et al., 2021). In addition, Fang Jie, a scholar in China, found in his study that callous-unemotional traits can mediate the relationship between childhood abuse and adolescent cyberbullying perpetration (Fang et al., 2020). Childhood abuse and cyberbullying victimization are individual adverse life experiences, and based on this study, the callous-unemotional traits are likely to play a moderating role between cyberbullying victimization and cyberbullying perpetration.

### 1.3 The moderating role of Internet morality

Internet morality is the moral values and principles of behavior that guide and regulate an individual’s online activities, including cyber ethical perceptions, emotions, and intentions (Ma and Lei, 2010). Previous studies have focused on moral excuses, such as the significant positive correlation between moral excuses and cyberbullying perpetration (Fang and Wang, 2020). Cyberbullying victimization can indirectly act on actual aggressive behavior through moral excuses (Xia and Sha, 2021). However, Internet morality belongs to the extension of traditional morality and has the role of subjective norms that can influence people’s behavior; in the network society, network morality can influence behavior (Jiang, 2023). Greene et al. (2008) put forward the dual processing theory that rational and perceptual processing work together with the individual’s ethical choices. The openness and anonymity of the Internet make it easier for individuals subjected to cyberbullying victimization to deal with the problem by adopting emotional processing, which breeds cyberbullying perpetration behavior. Empirical studies have also shown that Internet morality moderates the effects of violent environments on aggressive behavior (Jin et al., 2018) and the effect of pro-social behavior on cyber deviant behavior (Ma and Lei, 2010). This points out that individuals with high Internet morality can constrain their behavior to be ethical in the face of undesirable situations. In contrast, individuals with low Internet morality may exhibit cyber deviant behaviors, such as cyberbullying perpetration.

### 1.4 The co-moderating effects of callous-unemotional traits and Internet morality

Bosnjak et al. (2020) proposed the theory of planned behavior, which points out the complexities of human behavior and the possible moderating role of attitudes and subjective norms. The callous-unemotional personality traits can influence an individual’s attitudes and emotional perceptions (Xiao et al., 2014). In contrast, Internet morality is a unique form that reflects an individual’s subjective norms (Ma and Lei, 2010). There are four scenarios in the pathway of cyberbullying victimization influenced by cyberbullying perpetration: low callous-unemotional traits high Internet morality, high callous-unemotional traits low Internet morality, low callous-unemotional traits low Internet morality, and high callous-unemotional high Internet morality. According to the Theory of Planned Behavior, individual attitudes and subjective norms can moderate individual behavior. Whether there is a joint moderating effect between the two still needs to be further explored.

**FIGURE 1:**
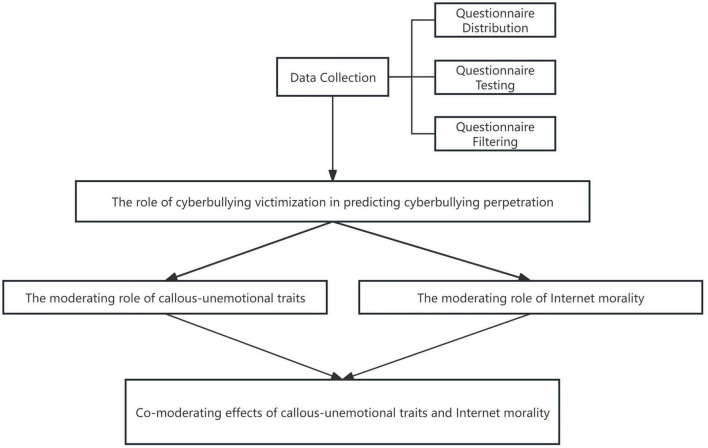
Research flowchart.

To summarize, based on China’s unique national conditions, this study takes college students who are active users of the Internet in the youth group as the research object and explores whether there is the “evil inversion” phenomenon of cyberbullying victimization to cyberbullying perpetration in the Internet from the perspective of criminal psychology and in combination with the general attack model. At the same time, the theory of planned behavior was expanded to include attitudes and subjective norms as moderating variables of individual behavior, exploring the moderating role of callous-unemotional traits and Internet morality. The study flow is shown in Figure 1. In total, the following hypotheses were formulated for this study:

H1: Cyberbullying victimization has a positive relationship with cyberbullying perpetration;

H2: The callous-unemotional traits can moderate the relationship between cyberbullying victimization and cyberbullying perpetration;

H3: Internet morality moderates the relationship between cyberbullying victimization and cyberbullying perpetration;

H4: The callous-unemotional traits and Internet morality can jointly mediate the relationship between cyberbullying victimization and cyberbullying perpetration.

## 2 Materials and methods

### 2.1 Participants and procedure

The convenience sampling method was used to randomly select college students from several colleges and universities in Beijing as the study population. The Criminology Ethics Committee of the People’s Public Security University of China reviewed the study. The survey used an online questionnaire to obtain data. The link to the questionnaire was sent to the participants by the instructor of the course, and the participants completed the questionnaire independently. The first part of the questionnaire informed the subjects of the purpose of the study and assured them that personal information would be kept strictly confidential. According to the formula *N* = *Z*^2^ × *P* (1 − *P*)/*E*^2^, *N* is the sample size, *Z* is the statistic, *P* is the probability value, and *E* is the allowed error. In this study, *Z* = 1.96 [95% confidence interval (95% CI)], *E* = 0.05, and *P* = 0.86 (Yang et al., 2022) give a required sample of no less than 185 subjects.

Therefore, in this study, 668 undergraduates from a university in Beijing were selected as the research subjects by convenience sampling method. The questionnaires were distributed through the online platform for administering the test, and a total of 633 valid questionnaires were obtained after removing invalid questionnaires, such as missing values in the data analysis and samples with answer times less than 60 s or greater than 600 s, with an effective recovery rate of 94.76%. Among them, the age range was 18–22, and the average age was 19.98 ± 1.24. The subjects were all non-rural college students with more than 1 year of exposure to the Internet. Statistics on specific demographic variables are shown in Table 1.

**TABLE 1:** Demographic variables.

Variant	Categorization	Frequency	Percentage
Gender	Male	331	52.29
	Female	302	47.71
Grade	First-year students	172	27.17
	Sophomores	233	36.81
	Juniors	101	15.96
	Seniors	127	20.06

### 2.2 Measures

#### 2.2.1 Cyberbullying victimization

We measured subjects’ level of cyberbullying victimization using the Cyberbullying Victimization Questionnaire (Lei et al., 2015). This nine-question questionnaire investigates, in a single dimension, the frequency of whether subjects have been subjected to property bullying, abusive venting, harassment, cell phone harassment, stalking, stigmatization, camouflage, privacy dissemination, and ostracism in the Internet in the past 3 months in nine areas. A 5-point scale was used from 1 (none) to 5 (several times a week). The Chinese version of this questionnaire showed good applicability. The internal consistency reliability coefficient was 0.91. Validated factor analysis showed good construct validity of the scale (GFI = 0.99, CFI = 0.99, AGFI = 0.98, RMSEA = 0.056).

#### 2.2.2 Cyberbullying perpetration

We used the revised Cyberbullying Perpetration Questionnaire (Lei et al., 2015), which showed good applicability in Chinese college students. The questionnaire consisted of 7 questions and was scored on a 5-point Likert scale from 1 (never) to 5 (always). The alpha coefficient of the scale in this study was 0.92. Validated factor analysis showed good scale construct validity (GFI = 0.96, CFI = 0.98, AGFI = 0.95, RMSEA = 0.060).

#### 2.2.3 The callous-unemotional traits

We used a revised version of the Callous-unemotional Traits Scale (Liu, 2018). The Chinese version of the scale has shown good applicability in the Chinese college student population. The scale is categorized into three dimensions: coldness, indifference, and callousness, with 21 questions. A 4-point scale ranged from 0 (non-compliant) to 3 (fully compliant). The alpha coefficient of the scale in this study was 0.72. Validated factor analysis showed good construct validity of the scale (GFI = 0.94, CFI = 0.95, AGFI = 0.93, RMSEA = 0.036).

#### 2.2.4 Internet morality

The Internet Morality Questionnaire was developed by Luo (2007), which includes four dimensions with a total of nine questions, namely, Internet morality Cognition, Internet morality Emotions, Internet morality Evaluation, and Internet morality Behavior, and scored on a 7-point scale ranging from −3 (totally disagree) to 3 (totally agree). The questionnaire was tested and shown to apply to the Chinese college student population. Validated factor analysis showed good construct validity of the scale (GFI = 0.97, CFI = 0.96, AGFI = 0.95, RMSEA = 0.053).

### 2.3 Data analysis

In this study, SPSS 26.0 was used for data entry and processing. Firstly, the Harman single-factor test was used for common method bias. Secondly, descriptive statistics were performed for the four variables, and the reliability of the scales was assessed using Cronbach’s alpha coefficient to assess the reliability of the scales. Partial correlation coefficients were calculated to test the relationship between the variables. In addition, covariance diagnostics were performed to confirm the absence of multicollinearity between variables and conformity to normal distribution. Finally, Hayes’s (2017) Process macro was used to test for moderating effects.

## 3 Results

### 3.1 Common method bias

This study was examined using the Harman single-factor test (Zhou and Long, 2004), and the results showed 13 factors with eigenvalues greater than 1. The variation explained by the first factor was 19.44%, less than the critical criterion of 40%, indicating no significant standard method bias in this study.

### 3.2 Mean number, standard deviation, and correlation analysis of variables

Table 2 presents the results of the descriptive statistics and correlation analysis in this study. The results indicated (as shown in Table 2) that cyberbullying victimization was significantly and positively correlated with cyberbullying perpetration and callous-unemotional traits; cyberbullying perpetration was significantly and positively correlated with callous-unemotional traits. The correlation between Internet morality and other variables was not significant.

**TABLE 2:** Mean number, standard deviation, and correlation analysis of variables.

Variables	*M* ± SD	1	2	3	4
1. Cyberbullying victimization	2.23 ± 1.24	1			
2. Cyberbullying perpetration	2.23 ± 1.27	0.73***	1		
3. The callous-unemotional traits	2.56 ± 0.75	0.69***	0.68***	1	
4. Internet morality	4.54 ± 1.44	0.32	−0.31	−0.29	1

***p < 0.001.

### 3.3 The role of cyberbullying victimization in predicting cyberbullying perpetration

The study demonstrates that the direct predictive effect of traditional bullying on cyberbullying perpetration is moderated by gender, with the predictive effect being significant among boys and not among girls (Zhu et al., 2019; Leban and Gibson, 2020). Meanwhile, age was correlated with both cyberbullying perpetration and cyberbullying victimization (Xie et al., 2022). Therefore, in order to further verify the relationship between cyberbullying victimization and cyberbullying perpetration, regression analysis was conducted controlling for gender and grade, and the analysis results are shown in Table 3. After controlling for gender and grade variables, cyberbullying victimization had a significant positive relationship with cyberbullying perpetration (β = 0.94, *t* = 69.54, *p* < 0.001), verifying Hypothesis 1. This suggests that after excluding other possible influences, there is still a positive relationship between cyberbullying victimization and perpetration, but what factors will be moderated still needs to be further explored.

**TABLE 3:** The relationship of cyberbullying perpetration with cyberbullying victimization.

Variant	β	*t*	*R* ^2^	*F*
Gender	−0.01	−0.25	0.10	32.41
Grade	0.03	2.35*		
Cyberbullying victimization	0.94	69.54***	0.90	150.98***

*p < 0.1, ***p < 0.001.

### 3.4 Moderating effects test

#### 3.4.1 Callous-unemotional traits and the test of Internet morality moderating effect

Process macros (Model 1) were used to test the moderating role of callous-unemotional traits and Internet morality in the optimistic relationship of cyberbullying perpetration behavior by cyberbullying victimization, respectively. In order to reduce the effect of multicollinearity among variables, each variable was standardized before entering the regression analysis, and the results are shown in Table 4, where the dependent variables of Model 1 ∼ Model 4 were all cyberbullying perpetration.

**TABLE 4:** Callous-unemotional traits and Internet morality moderating affect test results.

Variant	Model 1		Model 2		Model 3		Model 4	
	β	*t*	β	*t*	β	*t*	β	*t*
Gender	0.01	0.07	−0.01	−0.11	−0.01	−0.20	−0.01	−0.05
Grade	0.04	2.83**	0.04	3.09**	0.03	2.49*	0.04	2.72**
Cyberbullying victimization	0.90	49.82***	0.30	2.30***	0.94	66.97***	0.67	7.38***
Callous-unemotional traits	0.06	3.17**	−0.23	−3.64***				
Cyberbullying victimization × callous-unemotional traits			0.82	4.70***				
Internet morality					0.01	0.40	−0.10	−2.66
Cyberbullying victimization × Internet morality							0.31	3.00***
*R* ^2^	0.90		0.90		0.89		0.89	
*F*	137.15***		113.79***		134.77***		109.37***	

*p < 0.1, **p < 0.01, ***p < 0.001.

Model 1 and Model 2 show the results of the moderating effect test for the callous-unemotional traits. The data showed that cyberbullying victimization was a significant positive predictor of cyberbullying perpetration after controlling for gender and grade (β = 0.30, *t* = 2.30, *p* < 0.001), which further verified Hypothesis 1: the interaction term between the callous-unemotional traits and cyberbullying victimization had a significant positive effect on cyberbullying perpetration (β = 0.82, *t* = 4.70, *p* < 0.001), suggesting that the moderating effect of the callous-unemotional traits was significant, and Hypothesis 2 was verified. Model 3 and Model 4 show the test results of the moderating effect of Internet morality. The data show that cyberbullying victimization has a significant positive effect on cyberbullying perpetration (β = 0.67, *t* = 7.38, *p* < 0.001); the interaction term between cyberbullying victimization and Internet morality has a significant positive effect on cyberbullying perpetration (β = 0.31, *t* = 3.00, *p* < 0.001), which indicates that the moderating effect of Internet morality is significant, and Hypothesis 3 is verified.

The above findings suggest that callous-unemotional traits and Internet morality can play a moderating role in the relationship between cyberbullying and cyberbullying. To further explore how these two variables play a role in the relationship between cyberbullying victimization and perpetration, simple slope analyses were conducted to differentiate between high and low levels by adding or subtracting 1 SD from the mean.

**FIGURE 2:**
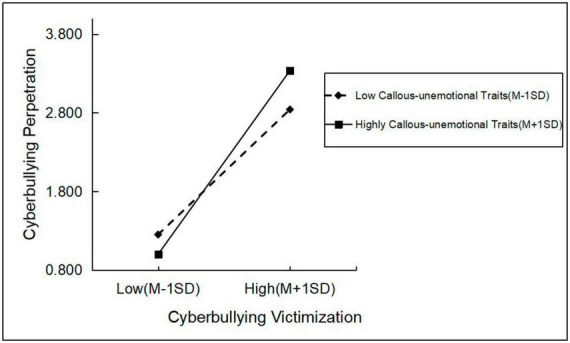
Moderating role of the callous-unemotional traits between cyberbullying victimization and cyberbullying perpetration.

**FIGURE 3:**
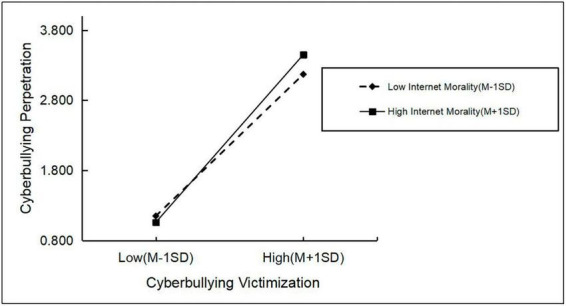
The moderating role of Internet morality between cyberbullying victimization and cyberbullying perpetration.

Table 5 and Figure 2 show that cyberbullying victimization positively predicted cyberbullying perpetration in both the high callous-unemotional traits group [*Simple slope* = 0.76, *p* < 0.001, 95% bootstrap CI (0.93, 0.99)] and the low callous-unemotional traits group [*Simple slope* = 0.58, *p* < 0.001, 95% bootstrap CI (0.56, 0.78)] and that the high callous-unemotional traits group had a more substantial positive predictive effect, further validating Hypothesis 2. Compared to Internet users with low callous-unemotional traits, Internet users with high callous-unemotional traits are likely to adopt more serious cyberbullying behaviors after being cyberbullied. Table 6 and Figure 3 show that cyberbullying victimization positively predicted cyberbullying perpetration in both the high Internet morality group [*Simple slope* = 0.67, *p* < 0.001, 95% bootstrap CI (0.95, 1.02)] and the low Internet morality group [*Simple slope* = 0.51, *p* < 0.001, 95% bootstrap CI (0.76, 0.92)] and that the high Internet morality group had a more substantial positive predictive effect as the degree of cyberbullying victimization increased, further validating Hypothesis 3. Internet users with high Internet morality are more likely to bully others after being bullied online than those with low Internet morality.

**TABLE 5:** Simple slope analysis of the callous-unemotional traits.

Level of moderating variables	Regression coefficient	SE	*t*	95% CI	
Average value	0.82	0.03	30.46***	0.77	0.88
Highly callous-unemotional traits (+1 SD)	0.97	0.02	44.57***	0.93	0.99
Low callous-unemotional traits (−1 SD)	0.67	0.06	12.13***	0.56	0.78

***p < 0.001.

**TABLE 6:** Simple slope analysis of Internet morality.

Level of moderating variables	Regression coefficient	SE	*t*	95% CI	
Average value	0.91	0.02	45.55***	0.87	0.95
High Internet morality (+1 SD)	0.99	0.02	56.45***	0.95	1.02
Low Internet morality (−1 SD)	0.84	0.04	20.45***	0.76	0.92

***p < 0.001.

#### 3.4.2 A test of the co-regulatory effect of the callous-unemotional traits and Internet morality

Stratified regression was used to test the co-moderating effect of the callous-unemotional traits and Internet morality on the relationship between cyberbullying victimization and cyberbullying perpetration (Wang Y. L. et al., 2020). The results (Table 7) showed that the triple interaction term was a significant positive predictor of cyberbullying perpetration (β = 0.59, *t* = 2.95, *p* < 0.05) after controlling the variables for gender and grade, which led to the conclusion that callous-unemotional traits and Internet morality play a co-moderating role in cyberbullying victimization’s optimistic relationship of cyberbullying perpetration, verifying Hypothesis 4.

**TABLE 7:** Results of the test of the co-moderating effect of the callous-unemotional traits and Internet morality.

Move	Variant	β	*t*	*R* ^2^	*F*
1	Gender	0.12	3.21	0.09	32.41
	Grade	0.26	6.85**		
2	Cyberbullying victimization	0.88	44.99***	0.90	111.12***
	The callous-unemotional traits	0.11	4.25*		
	Internet morality	−0.06	−2.84		
3	Cyberbullying victimization × the callous-unemotional traits	0.16	5.32***	0.90	74.35***
	Cyberbullying victimization × Internet morality	−0.45	−3.07*		
	The callous-unemotional trait × Internet morality	0.45	3.07**		
4	Cyberbullying victimization × the callous-unemotional traits × Internet morality	0.59	2.95*	0.91	66.13***

*p < 0.05, **p < 0.01, ***p < 0.001.

**FIGURE 4:**
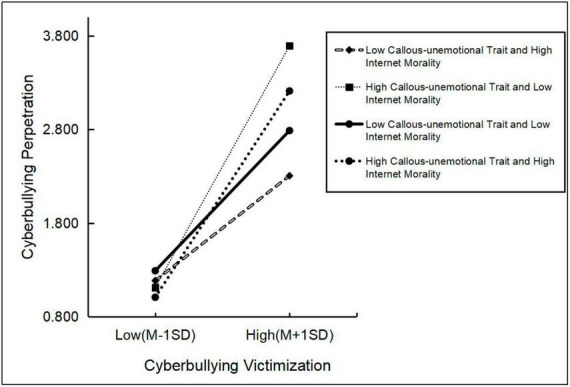
Co-moderating role of the callous-unemotional traits and Internet morality between cyberbullying victimization and cyberbullying perpetration.

A simple slope test was used to further explore the co-moderating role of callous-unemotional traits and Internet morality in the effect of cyberbullying victimization on cyberbullying perpetration, and the moderating effect plot is shown in Figure 4. According to the level, the group was categorized into low callous-unemotional traits and high Internet morality group (*Simple slope* = 0.43, *p* < 0.001), high callous-unemotional traits and low Internet morality group (*Simple slope* = 0.83, *p* < 0.001), low callous-unemotional traits and low Internet morality group (*Simple slope* = 0.39, *p* < 0.001), and high callous-unemotional traits and high Internet morality group (*Simple slope* = 0.76, *p* < 0.001). As can be seen from the figure, cyberbullying victimization has a significant positive relationship with cyberbullying perpetration at all four levels, and cyberbullying victimization has the most substantial positive relationship with cyberbullying perpetration at the high callous-unemotional traits at the low Internet morality level. This suggests that high callous-unemotional traits and low Internet morality are essential factors influencing the relationship between cyberbullying victimization and perpetration, and it has important implications for our future targeted recommendations.

## 4 Discussion

This study examined the moderating role of callous-unemotional traits and Internet morality in the relationship between cyberbullying victimization and perpetration. The study showed that cyberbullying victimization was a significant positive predictor of cyberbullying perpetration, controlling for gender and grade level. Whereas callous-unemotional traits and Internet morality not only played a moderating role in it separately but could also co-moderate the relationship between cyberbullying victimization and cyberbullying perpetration, the co-moderating effect was more substantial for low callous-unemotional traits and high Internet morality.

### 4.1 The impact of cyberbullying victimization on cyberbullying perpetration

The results indicated that cyberbullying victimization positively predicted cyberbullying perpetration, controlling for gender and grade variables, consistent with previous research findings (Kowalski et al., 2014; Wang, 2020; Zhang et al., 2022; Kim and Lee, 2023). The phenomenon of “evil reversal,” in which Internet users transform from bullied groups to bullies in cyberspace, is also verified. Agnew’s (1992) Social Learning Theory points out that aggressive behavior can be learned through external stimulation like other societal behaviors. Cyberbullying victimization is an extension of traditional bullying behavior (Wang, 2020). Through the learning of cyberbullying perpetration, Internet users in the context of cyberbullying victimization produced “an eye for an eye, a tooth for a tooth.”

Meanwhile, in frustration-induced aggression, the smaller the punishment expected by the aggressor, the greater the likelihood of practicing the aggressive behavior (Gilbert and Bushman, 2020). The anonymity of the Internet makes it less costly for Internet users to interact with each other, with little time and space constraints. It reduces the expected cost of punishment (Zhang et al., 2021), exacerbating the shift from cyberbullying victimization to cyberbullying perpetration.

### 4.2 The moderating role of callous-unemotional traits and Internet morality

#### 4.2.1 The moderating role of callous-unemotional traits

The callous-unemotional trait is a personality tendency that predisposes to violent crime, and people with the callous-unemotional traits are more aggressive and prone to antisocial behavior (Xiao et al., 2014). The present study shows that the callous-unemotional traits moderate the positive predictive effect of cyberbullying victimization on cyberbullying perpetration. This result suggests that callous-unemotional personality traits can exacerbate the shift from cyberbullying victimization to cyberbullying perpetration, validating the general attack model. In discussing the effects of the callous-unemotional traits on juvenile violent offenders, Yang and Huang (2013) emphasized that the callous-unemotional traits have a positive predictive effect on premeditated sexual violence. Liu (2018) also showed that the callous-unemotional traits did not predict reactive aggression in her revision and initial application of the Callous-unemotional Traits Scale. The cyberbullying perpetration behavior in this study was influenced by cyberbullying victimization. It was impulsive violence or reactive aggression, in which the role of the callous-unemotional traits was inconsistent with previous studies. This may be because the present study used the callous-unemotional traits as a moderating variable, which acted in conjunction with the interaction term for cyberbullying perpetration. In addition, the low cost of engaging in aggression in the online environment facilitates the aggressive behavior of Internet users with highly callous-unemotional. Although the callous-unemotional traits have strong stability and genetic likelihood (Xiao et al., 2014), with the depth of research, some scholars have found that environmental factors are also able to influence the changes in the level of the callous-unemotional traits (Frick and White, 2008). This suggests that in the future, we can avoid the phenomenon of online “evil reversal” by influencing the callous-unemotional traits of Internet users.

#### 4.2.2 The moderating role of Internet morality

Internet morality is an extraordinary internal norm, a code of conduct for online social relationships (Luo, 2007). This study found that Internet morality significantly moderates the relationship between cyberbullying victimization and cyberbullying perpetration among Internet users. Internet morality and real-society morality are universal and particular, and behavior on the Internet will be naturally influenced by morality on the Internet. An individual’s level of Internet morality can influence the formation of cyberbullying perpetration. The analysis of the moderating effect of Internet morality shows that high Internet morality can inhibit the transition from cyberbullying victimization to cyberbullying perpetration behavior when subjected to less cyberbullying perpetration, which is consistent with the results of existing studies (Jin et al., 2018). However, as the level of cyberbullying victimization increases, individuals with high Internet morality instead have a more substantial moderating effect on the transformation of cyberbullying victimization to cyberbullying perpetration, which may be because Internet morality consists of the four dimensions of moral cognition, moral emotion, moral evaluation, and moral behavior (Luo, 2007) while being cyberbullied may have multiple effects on the cognitive, emotional, and behavioral dimensions of the bullied individual (Xiao et al., 2022), and the mediating effects of the various dimensions of Internet morality in it can be explored in the future.

#### 4.2.3 Co-moderating effects of callous-unemotional traits and Internet morality

In the mechanism by which cyberbullying victimization affects cyberbullying perpetration, callous-unemotional and Internet morality regulate the relationship between the two and play a co-regulatory role. In the process of “evil reversal,” the victim cannot get timely and effective help after being violated, falls into a negative psychological state, and produces a malignant transformation of psychology and behavior under the joint action of multiple factors of oneself and the environment (Guo, 1997). As for the “evil reversal” in the network, this study, based on the Theory of Planned Behavior, confirms that callous-unemotional as a personality trait and Internet morality as a reflection of individual subjective norms can jointly influence the formation of cyberbullying penetration. The results of the simple slope test show that under the level of high callous-unemotional and low Internet morality, cyberbullying victimization is the most significant positive predictor of cyberbullying perpetration, and low callous-unemotional and high Internet morality can effectively inhibit the transformation of cyberbullying victimization to cyberbullying perpetration, which is consistent with the results of existing studies (Jin et al., 2018; Wang et al., 2021; Zhang et al., 2023). In addition, moral excuses theory states that moral excuses are correlated with the emergence of aggressive behavior (Bandura, 2014). On the one hand, moral shirking can be affected by individual personality traits, and individuals high in callous-unemotional traits have difficulty distinguishing between moral violations and customary violations and are more likely to act in a way that harms others (Wang et al., 2021). On the other hand, individuals high in Internet morality can rationally analyze the possible adverse consequences of aggressive behaviors, inhibiting cognition and preventing aggressive behaviors (Jin et al., 2018). The effect of the two of them on aggressive behavior is consistent with the results of this study, which validates the theory of moral excuses.

### 4.3 Research implication

This study has the following innovations. Theoretically, the study starts from the criminological phenomenon of “evil reversal,” confirms the phenomenon of vicious transition from cyberbullying victimization to cyberbullying perpetration on the Internet, and verifies the moderating role of callous-unemotional and Internet morality, which provides a new research perspective for criminal psychology. Meanwhile, previous studies have mainly studied the callous-unemotional traits as a mediating variable, ignoring the fact that personality traits are also an important moderating variable, based on which the present study confirms that the callous-unemotional traits can play a moderating role in the relationship between cyberbullying victimization positively predicting cyberbullying perpetration.

In practice, the findings confirm, cyberbullying victimization has a positive relationship with cyberbullying perpetration. In order to effectively avoid vicious transformations, the speech of Internet users can be regulated by implementing a real-name system for online communities and strengthening accountability. At the same time, it is recommended that Internet platforms establish an early warning and monitoring system to protect the health of the Internet ecosystem. On the other hand, the study also confirm that either low callous-unemotional traits or high Internet morality are a protective factor that prevents cyberbullying victimization from shifting to cyberbullying perpetration, which provides a reference for future interventions in the cyber “evil reversal” process. For the problem of cold-hearted traits predisposing college students to emotional processing difficulties, Lochman and Wells (2004) proposed the Coping Power Program to manage anger, which includes controlling the degree of arousal to provocation, correcting hostile attributional biases, and practicing anger management. Therefore, we can alleviate the emotional processing deficits of college students’ cold-hearted traits by incorporating a coping power program into their daily Internet use. It also suggests that the cyberbully may be the last cyberbullied person, and the cyberbullied person may be the next. It makes cyberbullying spread, which in turn leads to a ring of more severe consequences. Teachers and parents should strengthen the education of adolescents’ moral cognition, promote their correct understanding of moral phenomena and behaviors on the Internet, and cultivate their positive cyber moral intention to effectively avoid the vicious transformation from cyberbullying victimization to cyberbullying perpetration, and especially for young people who have been subjected to cyberbullying, help them realize that cyberbullying is inherently wrong.

### 4.4 Limitations and further work

This study also has specific areas for improvement. Firstly, in terms of sample selection, scholars Velicer and Fava (1998) found that in exploratory factor analysis, the number of samples is an essential variable in determining a good model. While the sample of this study is limited, and all of them are urban college students, the sample could be more representative. In the future, the relationship between cyberbullying victimization and cyberbullying perpetration can be further explored by increasing the sample of subjects. Secondly, the use of cross-sectional studies has some limitations in explaining the intrinsic causal relationship, and longitudinal studies need to be used in the future to explore the mechanism of the transition from cyberbullying victimization to cyberbullying perpetration. In the selection of variables, Olweus (2012) confirms the correlation between cyberbullying and traditional bullying, that cyberbullying was not a significant predictor of self-esteem after controlling for the effects of traditional bullying. However, some studies have also confirmed that cyberbullying can positively predict deviant behavior even after controlling for traditional bullying (Li et al., 2015). Meanwhile, it is worth exploring whether there is a bidirectional role between cyberbullying and cyberbullying. In the future, a comparative study between traditional and cyberbullying and bullied behaviors can be conducted to improve the cyclic processing model of bullying continuously. In terms of transformational mechanisms, this study focuses on the moderating role of individual personality traits and subjective norms on aggressive behavior. However, there may be other moderating variables from an individual’s perspective—self-control (Li et al., 2015), gender (Zhu et al., 2019), emotion regulation strategies (Wang, 2020), and so on. At the same time, environmental input variables can alter an individual’s cognition and emotions, affecting behavioral intentions and thus leading to aberrant behavior (Anderson and Bushman, 2002). For example, in a study on the effects of parental head-down behavior on adolescent cyberbullying and bullying, it was shown that adolescent parent-child intimacy and internalization problems jointly affect cyberbullying, cyberbullying under the effect of parental head-down behavior (Liu and Chen, 2023). Environmental factors such as poor peer relationships (Eisenhut et al., 2023) and parenting styles (Zhang and Zhu, 2023) can be added to future studies for research. Finally, this study only confirms the existence of the phenomenon of cyberbullying to cyberbullying in cyberspace. In the future, we can also propose targeted measures from the perspective of criminal psychology to guide practice with theory better.

## 5 Conclusion

The following conclusions were obtained in this study: (1) controlling for gender and grade, cyberbullying victimization has a positive relationship with cyberbullying perpetration; (2) callous-unemotional traits can modulate the relationship between cyberbullying victimization and cyberbullying perpetration, and cyberbullying victimization has a more substantial positive relationship on cyberbullying perpetration at high callous-unemotional traits; (3) Internet morality can modulate the relationship between cyberbullying victimization and cyberbullying perpetration, and as the level of cyberbullying victimization increases, high Internet morality has a more substantial moderating effect; (4) callous-unemotional and Internet morality can co-regulate the relationship between cyberbullying victimization and cyberbullying perpetration, with a more substantial co-regulatory effect for low callous-unemotional traits high Internet morality.

## Data availability statement

The original contributions presented in this study are included in the article/supplementary material, further inquiries can be directed to the corresponding author.

## Ethics statement

The studies involving humans were approved by the Criminology Ethics Committee of the People’s Public Security University of China. The studies were conducted in accordance with the local legislation and institutional requirements. The participants provided their written informed consent to participate in this study.

## Author contributions

WZ: Writing – original draft. JS: Writing – review & editing.
